# Release of Extracellular Matrix Components after Human Traumatic Brain Injury

**DOI:** 10.1523/ENEURO.0488-24.2025

**Published:** 2025-06-19

**Authors:** Michael Bambrick, Deena Godfrey, Mark D. Johnson, Jeffrey D. Esko, Biswa Choudhury, Alejandro Gomez Toledo, Mousumi Paulchakrabarti, Carla Fortes, Kevin J. Staley, Ann-Christine Duhaime

**Affiliations:** ^1^ Department of Neurosurgery, Massachusetts General Hospital, Harvard Medical School, Boston, Massachusetts 02114; ^2^ Department of Physical Medicine and Rehabilitation, Spaulding Rehabilitation Hospital, Harvard Medical School, Charlestown, Massachusetts 02129; ^3^ Department of Neurosurgery, University of Massachusetts Memorial Health, University of Massachusetts Chan Medical School, Worcester, Massachusetts 01655; ^4^ Department of Cellular and Molecular Medicine, University of California, San Diego Medical School, La Jolla, California 92093; ^5^ Department of Neurology, Massachusetts General Hospital, Harvard Medical School, Boston, Massachusetts 02114

**Keywords:** cerebral spinal fluid, extracellular matrix, sulfated glycosaminoglycans, traumatic brain injury

## Abstract

Animal studies and human tissue experiments have demonstrated that traumatic brain injury (TBI) causes damage to the extracellular matrix (ECM). To test the hypothesis that TBI causes disruption of sulfated glycosaminoglycan (sGAG) in the ECM, we measured levels of sGAG in the cerebrospinal fluid (CSF), blood, and urine, in patients with severe TBI in the acute postinjury period. Samples of CSF, blood, and urine were obtained within 72 h of injury in patients who received external ventricular drains as part of their treatment of severe TBI. Levels of chondroitin and heparan sGAGs were measured, along with their disaccharide constituents. Demographic information, presence of polytrauma, brain injury load, and distance of radiologically visible parenchymal injury from the ventricle were analyzed for correlation with total subtype sGAG levels. Levels were measured in 14 patients ranging in age from 17 to 90 years. CSF sGAG levels were variable among patients, with higher sGAG levels in plasma compared with CSF. Patients with polytrauma had nonsignificantly higher blood sGAG compared with patients with isolated head injury. Subcategories of CSF sGAG levels correlated with distance from the ventricle of parenchymal injury but not with brain injury load. This study is the first to measure sGAG levels in ventricular CSF and the first to analyze levels in TBI. These data demonstrate the elevation locally of intracranial sGAGs after severe TBI and suggest rapid local metabolism of these breakdown products. The consequences of ECM breakdown may provide unique therapeutic and preventive avenues to mitigate postinjury sequelae.

## Significance Statement

The extracellular matrix (ECM) is what makes a solid organ solid and comprises ∼20% of the brain's volume. In humans, the effect of severe traumatic brain injury (TBI) on the ECM has not previously been investigated, including what happens to GAGs and proteoglycan fragments after trauma. We investigated if glycosaminoglycan levels in CSF, blood, and urine could be used as biomarkers after severe TBI in humans. The results demonstrated that specific intracranial sGAGs are elevated locally after severe TBI and appear to be rapidly metabolized. Further investigation of ECM breakdown products may help with the treatment and prevention of TBI complications such as early edema and late posttraumatic epilepsy.

## Introduction

**Figure 1.**
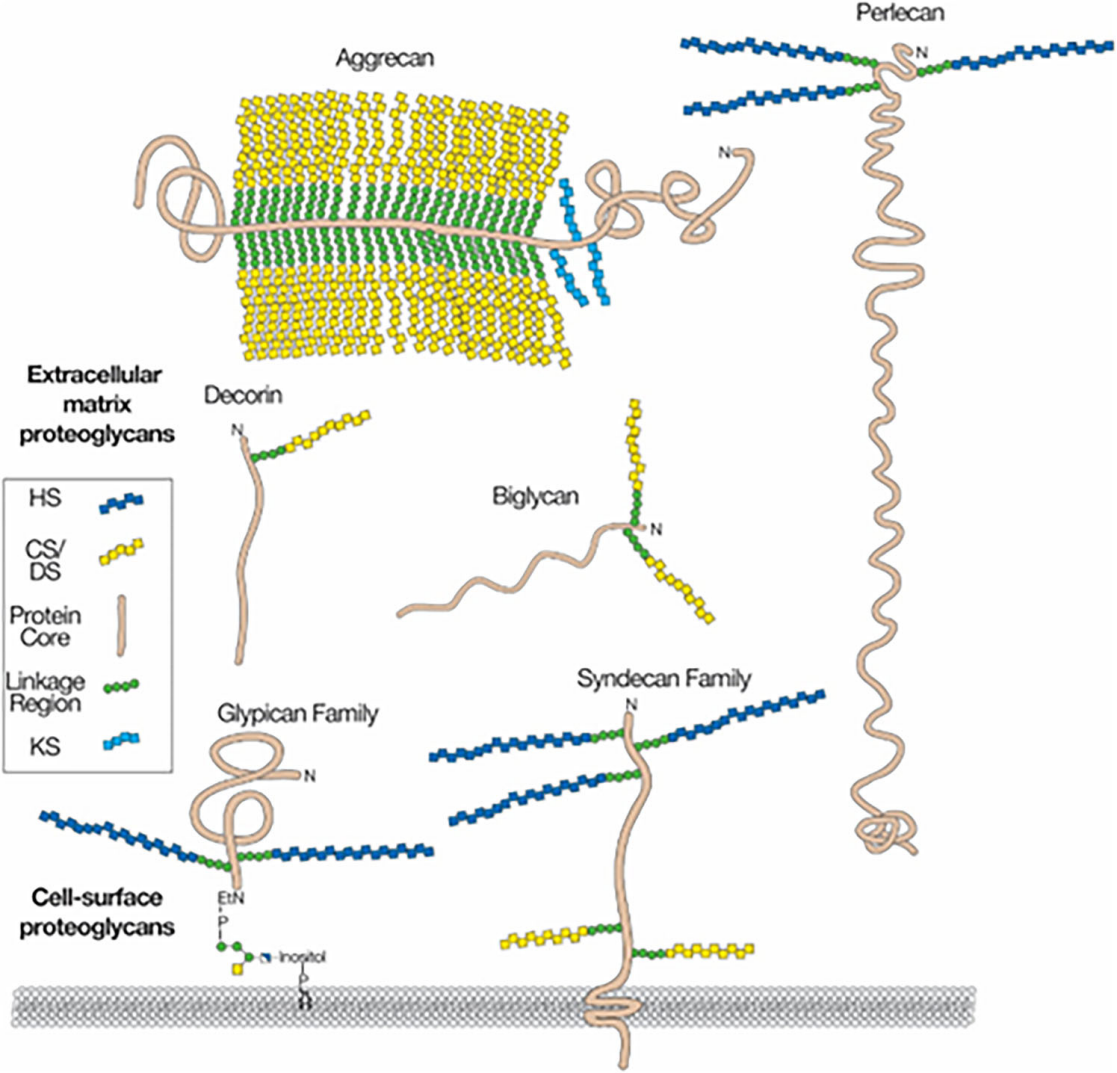
Proteoglycans and sulfated glycosaminoglycans. Proteoglycans consist of a protein core (brown) and one or more covalently attached glycosaminoglycan chains (dark blue, HS, heparan sulfate; yellow, CS/DS, chondroitin sulfate/dermatan sulfate; light blue, KS, keratan sulfate). Membrane proteoglycans either span the plasma membrane (Type I membrane proteins) or are linked by a glycosylphosphatidylinositol (GPI) anchor. Extracellular matrix proteoglycans are usually secreted, but some proteoglycans can be proteolytically cleaved and shed from the cell surface (data not shown). Reprinted with permission from Merry et al. (2022). HS, heparan sulfate, CS/DS, chondroitin sulfate/dermatan sulfate, KS, keratan sulfate.

The ECM holds the component cells of an organ together and contributes to the solidity of an organ (Frantz et al., 2010). It fills the extracellular space (Hrabetova et al., 2018), comprising approximately 20% of the brain's volume (Syková and Nicholson, 2008; Tønnesen et al., 2018). The ECM is a hydrogel filled with extracellular fluid, proteoglycans [glycoproteins that contain long, polymeric sulfated glycosaminoglycans (sGAG)], and hyaluronan [hyaluronic acid; a nonsulfated glycosaminoglycan (GAG); Karamanos, et al., 2021; Fig. 1]. GAGs are assembled by the copolymerization of alternating residues of *N*-acetylglucosamine and glucuronic acid in heparan sulfate (HS) and hyaluronan, *N*-acetylglucosamine and galactose in keratan sulfate, and *N*-acetylgalactosamine and glucuronic acid in chondroitin sulfate (CS)/dermatan sulfate. The chains undergo variable modifications including deacetylation of *N*-acetylglucosamine residues in heparan sulfate, *O*-sulfation at various positions, and epimerization of glucuronic acid to iduronic acid. The disaccharide constituents of GAG are named based on a four-character code reflecting their specific structures, including numbers denoting their sulfation characteristics (disaccharide structure code; Lawrence et al., 2008a). In the brain, chondroitin sulfate and heparan sulfate are the most plentiful sGAGs, and hyaluronan is the most plentiful GAG (Miyata and Kitagawa, 2017). GAGs are further characterized by their disaccharide component characteristics, including the position and number of sulfate groups (Lawrence et al., 2008a). Disaccharide characteristics can vary among species and sites and during development and plasticity (Miyata and Kitagawa, 2017).

The sulfation of the disaccharides imparts a high negative charge density to the ECM (Lieleg et al., 2009; Morawski et al., 2015). These anatomically fixed charges dramatically reduce the diffusion of charged molecules through the ECM by electrostatic interactions (Syková and Nicholson, 2008; Lieleg et al., 2009). We have proposed that, just as in the intracellular space (Glykys et al., 2014; Rahmati et al., 2021), fixed charges in the extracellular space reduce the local concentration of extracellular chloride (Glykys et al., 2017; Epsztein et al., 2018; Normoyle et al., 2025). This alters the local chloride concentration gradients across the neuronal membrane. These gradients may alter the direction and effects of activation of local inhibitory GABA_A_ synapses. This is in keeping with the Donnan exclusion principle which accounts for the effect of an immobile ion on the development of mobile ions (Fatin-Rouge et al., 2003; Normoyle et al., 2025).

In rodent models, when neurons die, the surrounding ECM is replaced (Yi, et al., 2012; Cregg et al., 2014). After significant insults such as severe traumatic brain injury (TBI), large numbers of neurons die in a short period of time, and the metalloproteinases that break down the extracellular matrix are detectable in the CSF and blood (Grossetete et al., 2009; Zheng et al., 2013). The resultant (and possibly also directly traumatic) dissolution of the ECM and its component sulfated disaccharides should result in large influxes of chloride salts and water into the extra- and intracellular spaces (Glykys et al., 2017). These fluid shifts may contribute to acute morbidities of TBI such as cerebral edema, for which more effective prevention and therapies are still needed (Jha et al., 2019). Chronic increases in sGAGs could degrade inhibitory GABA_A_ chloride currents and contribute to posttraumatic epilepsy.

We know very little about dissolution and resorption of the brain's ECM under physiological or pathological conditions. Matrix metalloproteinases that are released and activated in response to injury hydrolyze the core proteins of the ECM, releasing GAGs (Verma and Hansch, 2007). Proteoglycans and GAGs are catabolized in a complex series of enzymatic reactions within lysosomes (Prabhakar and Sasisekharan, 2006) or by extracellular enzymes, such as heparanases or hyaluronidases. However, when neurons die acutely after TBI, it remains unknown what happens to GAGs and proteoglycan fragments. Catabolism of released GAGs by surviving neuronal, glial, and vascular cells may occur. However, after major injuries, there may be more GAGs released than the remaining capacity to catabolize them, and GAGs may disperse to the CSF and blood after TBI. GAG content and composition have neither been measured in ventricular CSF nor have they been studied as biomarkers after trauma (Huibregtse et al., 2021). In this study, we measured GAG levels in CSF, blood, and urine in human patients treated with ventriculostomies (external ventricular drains) for the management of increased intracranial pressure after TBI. We hypothesized that CSF levels of GAGs would be increased compared with historical data derived from lumbar puncture, that CSF levels of GAGs would increase with higher brain injury load or other injury characteristics, and that polytrauma patients might have higher blood and/or urine GAG levels compared with patients with isolated head injury.

## Materials and Methods

### Sample collection

The data presented are part of the Citizens United for Research in Epilepsy's multicenter research study. Two Level 1 trauma centers enrolled patients of either sex with severe acute traumatic brain injury (Glasgow Coma Scale ≤8) who required the placement of an external ventricular drain for clinical purposes. Ventriculostomies are placed at the treating team's discretion and in general are used in these two centers for patients with swelling-prone injuries to manage anticipated or ongoing elevations in intracranial pressure (Duhaime and Rindler, 2015). Patients with a pre-existing history of epilepsy or additional confounding neurologic conditions such as stroke, hydrocephalus, or neurodegenerative conditions were excluded.

All CSF and urine samples from the subjects were collected as waste material, either discarded extra fluids obtained during clinical sampling, or discarded fluids during routine care when drainage bags were replaced. Blood samples were obtained from clinical lab excess material. Samples from urine and blood were timed to be obtained as closely as possible to the time of CSF sampling, typically simultaneously or within hours. No excess samples were drawn for the purpose of the study, and no direct contact with patients/families was made by the investigators, who coordinated obtaining excess samples directly from the treating team or clinical labs. As a result, the Institutional Review Board granted a Waiver of Consent for the use of waste samples for the purpose of the study. At Site 1, access to complete medical records was available, and therefore additional information regarding extracranial injuries and neuroimaging was able to be analyzed.

Samples of urine and CSF were frozen and stored for later sGAG analysis, while the blood samples were centrifuged, and the separated plasma was extracted and frozen. These samples were obtained within the first 72 h of injury to standardize the time window as much as possible within the limitations of the Waiver of Consent guidelines. For patients who were transferred to our center in the first 1–2 d after injury or had a ventriculostomy placed in a delayed fashion, CSF samples were collected within 72 h of arrival or ventriculostomy placement making some initial sampling times up to 5 d postinjury. To assess changes over time, additional samples were acquired during subsequent 72 h time epoch windows if the ventriculostomy was still in place and draining.

The samples were analyzed at a separate site laboratory (Site 3). Samples were processed to analyze concentrations of sGAG subtypes chondroitin sulfate and heparan sulfate by digestion of the samples with chondroitinase ABC or heparin lyases and quantification of the disaccharide components as described below. CSF and urine (4 ml) were extracted, except for two of the samples in which only 2 ml was available. GAG isolation from 200 µl of plasma samples was diluted using 200 µl of ice-cold UltraPure DNase/RNase-free water. Then 375 µl of 2× pronase digestion buffer [50 mM sodium acetate (NaOAc) with 200 mM NaCl, pH 6.0] was added and mixed thoroughly. Protease [25 µl (0.5 mg), P5147, Sigma-Aldrich] was added, and the samples were incubated at 37°C for 16 h with vertical tumbling of the tubes.

### DEAE Sephacel chromatography for isolation of GAG

Protease-treated samples were loaded on a small disposable chromatography column packed with 0.3 ml of prewashed diethylaminoethyl (DEAE) Sephacel matrix. The prewashing of the DEAE Sephacel matrix was done using 2 ml of DEAE equilibration buffer (50 mM NaOAc with 200 mM NaCl and 0.1% Triton X-100, pH 6.0). Samples were loaded on the column and allowed to flow under gravity. GAG molecules are negatively charged and bind to the DEAE resin through charge interaction. The sample tube was rinsed with 1 ml of DEAE wash buffer to ensure complete transfer of the samples. DEAE column was washed with 6 ml (20× bed volume) of DEAE wash buffer, and finally the bound GAGs were eluted in 2.5 ml of DEAE elution buffer (50 mM NaOAc with 2 M NaCl, pH 6.0) in a 15 ml tube. Samples (2.5 ml) were loaded on conditioned PD10 desalting columns (10% ethanol/water) and allowed to flow through completely. Finally, GAGs were eluted using 3.5 ml of 10% ethanol solution and lyophilized.

### Heparan sulfate and chondroitin sulfate analysis

Lyophilized GAG underwent chondroitin sulfate (CS) and heparan sulfate (HS) digestion using chondroitinase ABC and heparinase I, II, and III. After digestion, the samples were purified using 3K spin filtration, tagged with [^12^C]aniline, mixed with [^13^C]aniline-tagged disaccharide standards, and analyzed by liquid chromatography/mass spectrometry (Lawrence et al., 2008b). We analyzed the distribution of component disaccharide species in these samples in order to assess the degree to which the released GAGs were sulfated, which might contribute to ionic shifts with physiologic consequences. The values for the individual disaccharides were summed to determine the total amount of GAG recovered in each sample.

### CT/MRI imaging analysis

Following collection of the samples, the subjects’ clinical imaging results per radiology reports and the images themselves were reviewed by a neurosurgeon with a background in standardized neurotrauma image analysis (ACD), blinded to GAG levels, to determine both the imaging-derived injury load and the distance of the closest visually damaged area to the lateral ventricle where the EVD was placed. The brain injury load was scored based on a summation of five different scores assigned to each patient (total range, 0–21 points; please refer to Table 1), using the National Institutes of Health Common Data Elements definitions for different types of traumatic lesions (Duhaime et al., 2010). Extradural injury (skull fracture or hemorrhage) was scored as 0 if absent and 1 if present. Supratentorial parenchymal hemorrhage/contusion was scored as present (1) or absent (0) in each lobe bilaterally, with scores thus ranging from 0 to 8. Subdural and/or subarachnoid hemorrhages were scored similarly by lobe, with possible scores also ranging from 0 to 8. Posterior fossa injury was scored as 0 if absent, 1 if parenchymal injury or subdural/subarachnoid hemorrhage was 1–2 cm in diameter, and 2 if larger areas were involved. An additional point was added for gross intraventricular hemorrhage (IVH larger than 5 mm) and another point for midline shift measured at the foramen of Monro ≥1 cm. Thus, using this system, the total injury load score had allowable values from 0 to 21. In addition to imaging load, the distance from the nearest parenchymal contusion/hemorrhage site to the lateral ventricle was determined by measuring from the edge of the lesion to the edge of the ventricle in millimeters. Brain injury load and distances were corroborated by a second, independent reviewer (M.B.). All images analyzed were obtained within the first 72 h of injury, occurring within the initial time epoch of sample collection.

**Table 1.** Image “injury load” scoring system

Imaging feature	Scoring	Range of points
Extradural injury (skull fracture or hematoma)	Absent = 0, present = 1	0–1
Supratentorial parenchymal hemorrhage	1 point for present in each lobe (right and left)	0–8
Subdural and/or subarachnoid hemorrhage	1 point for present over each lobe (right and left)	0–8
Posterior fossa hemorrhage (parenchymal, subdural, or subarachnoid)	1 point if 1–2 cm, 2 points if larger	0–2
Intraventricular hemorrhage >5 mm	1 point	0–1
Midline shift >1 cm	1 point	0–1
Total possible points		0–21

### Trauma classification and scoring

Subjects were classified as having isolated head trauma or multisystem trauma by records review. Patients with involvement of head only or head plus a single low-severity extremity injury were classified as predominantly isolated head injury, while those with more severe and multiregion injuries were classified as multisystem trauma. Injury body region distribution and severity were verified using the abbreviated injury score (AIS) and injury severity score (ISS) for each individual when available; these scores sum injury subscores for each body region. The severity of extracranial trauma was quantified by summing the AIS subscores and subtracting the score for head injury. The ISS is a more detailed score reflecting overall injury severity, including of the head, and also was analyzed for correlation with measured GAG values.

### Statistical analysis

Differences among levels of disaccharide subtypes were determined by ANOVA test and subsequent post hoc analysis to assess for the significance of differences. Injury-related variables, including the severity of extracranial trauma (AIS score values excluding the AIS head score), ISS, imaging load, proximity to the ventricle (as previously described), and age as a demographic variable, were presented as continuous variables. The correlation of individual sGAG results to injury-related variables and age was assessed using Pearson’s correlation coefficient equation, and subsequently *t* value was calculated. These were then used to calculate the *p*-value, and it was adjusted to reflect the number of independent tests performed. While there are no normative values for ventricular CSF GAGs from healthy patients, levels were compared with those obtained from lumbar puncture in healthy controls collected for other purposes, acknowledging potential confounding differences from these sources (discussed further below; Zhang et al., 2011; Hendriksz et al., 2015; Yu et al., 2019).

### Transparency, rigor, and reproducibility summary

The study design, variables, and analyses for this exploratory study were not preregistered. The sample size was based on admission patterns for the participating hospitals and extrapolated over the period of enrollment. Because this was a minimal-risk study of comatose patients, all patients who fulfilled the entry criteria were enrolled and had biofluids collected and analyzed. No enrolled subjects were excluded from the analysis. Subjects were unaware of assignment. Fluid biomarker measurements were performed by investigators blinded to the relevant characteristics of the participants. Samples were acquired during work hours between 2018 and 2020. Samples were stored at −80°F for up to 2 years prior to analysis. Samples were analyzed in two batches. No unexpected events occurred. The analyses of the glycosaminoglycans were validated for research use. The equipment and analytical reagents used to perform measurements on the fluid biomarkers are available via the UCSD Glycomics Center. The clinical criteria (e.g., primary diagnosis or prognostic factor) have not been established as standards in the field. The statistical tests used were based on normally distributed data. Corrections for multiple comparisons were applied. The data underlying the presented analyses are available on request. No private analytic code was used in the analyses. All biofluid samples used to conduct the study were obtained by the investigators, and none of the participants have provided permission to be re-contacted about the use of their samples for future research. The authors agree to provide the full content of the manuscript when permitted by the embargo policy of the Journal.

## Results

The 14 patients ranged in age from 17 to 90 years of age. Site 1 contributed 10 subjects, and Site 2 contributed 4 subjects. The mean time from injury to initial CSF sampling in those patients with exact times recorded was 74 h and 21 min (standard deviation of 23 h and 19 min; Table 2). Four patients also had samples in the 4–6 d postinjury epoch, and one patient had a third set of samples at 9 d after injury. Of the 10 Site 1 patients, who had full extracranial injury profiles, 4 had isolated TBI, and 6 had polytrauma. Of the polytrauma patients, extracranial injury severity (AIS minus head injury severity scores) ranged from 3 to 19 (mean, 9.8), reflecting a severity range from mild to critical thoracoabdominal, skeletal, and soft tissue injuries.

**Table 2.** Time (hours) from injury to first CSF sample collection for 9 subjects from Site 1

Subject	Time from Injury (hours)
1	71.98
2	46.83
3	38.43
4	64.38
5	70.07
6	85.95
7	89.58
8	86.83
9	115.05
Mean (SD)	74.35 (23.32)

Time from injury to initial CSF sample collection. Specific timing data available for 9 of the 10 subjects recruited from Site 1. CSF, cerebrospinal fluid; SD, standard deviation.

The collection of CSF from ventricular catheters/drainage reservoirs, as well as the collection of blood and urine samples from excess laboratory or drainage samples, was readily accomplished at both institutions with no identified risk to patients. No complications or limitations of these procedures were found. Storage, shipment, and remote analysis of samples were accomplished without losses, demonstrating that multicenter collection and analysis of ventricular CSF, blood, and urine for GAGs after severe head trauma could be executed as a minimal-risk prospective human trial in patients with traumatic coma.

For this report, we analyzed only the initial time point of sampling for each patient, within the first 72 h of injury. Several patients had more than one sample from a single time point because of large volumes of fluid (CSF, plasma, or urine) which were divided into aliquots and tested individually, resulting in 21 total samples from the 14 patients at the first sample epoch. Values from multiple samples from the same patient and time point were averaged for each patient.

### Chondroitin sulfate in ventricular CSF 

**Figure 2.**
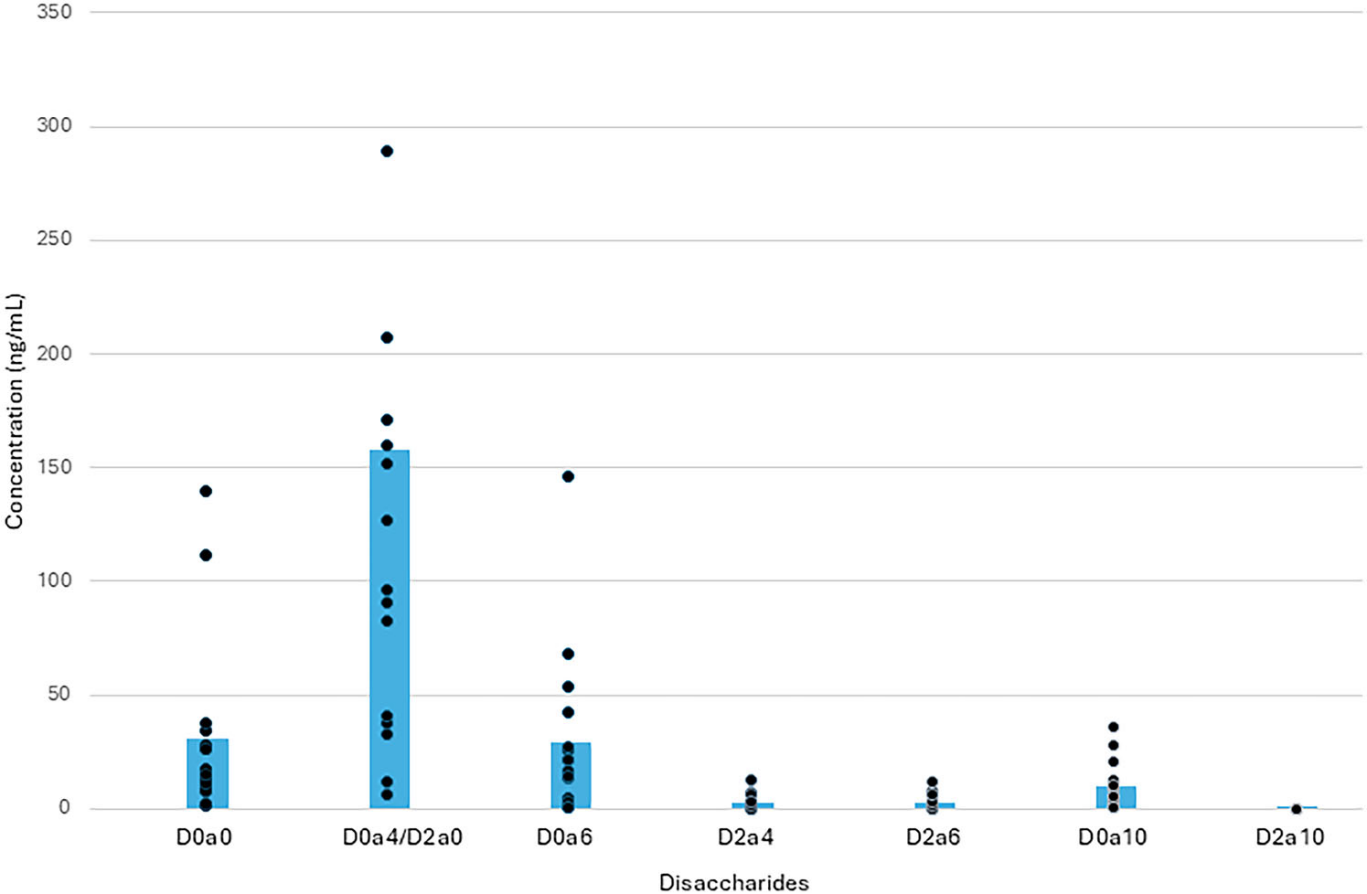
CSF chondroitin sulfate disaccharide concentration. Concentration of chondroitin sulfate disaccharides within cerebrospinal fluid samples. Concentration is measured in ng/ml with D0a4/D2a0 disaccharide showing the highest average concentration and a statistically significant difference from the rest of the disaccharides (*p* < 0.005).

The mean concentration of sulfated CS GAGS in the CSF was 218 ng/ml ± 224 (SD; Fig. 2). These results fall below the range for published mean levels in healthy pediatric patients from lumbar CSF samples done for negative meningitis or other types of evaluations, which had a mean of 670 ng/ml ± 570 ng/ml (Zhang et al., 2011). The individual disaccharides present in the analysis showed a nonuniform distribution, with D0a4/D2a0, a disaccharide with a single sulfate group, having the greatest concentration, with a mean of 149 ± 152 ng/ml. Analysis using ANOVA with a post hoc test showed that this disaccharide had significantly greater concentration than the rest of the disaccharides measured in the study, with a *p*-value that was <0.005 in relation to the other disaccharides. Among the other disaccharides, D0a0, which is not sulfated, had a mean concentration of 28 ± 36 ng/ml. D0a6, which has one sulfate group, had 26 ± 34 ng/ml. D2a4, which has two sulfate groups, had 2.6 ± 3 ng/ml. D2a6, also having two sulfate groups, had 2.6 ± 3 ng/ml. D0a10, with two sulfate groups, had 9.6 ± 9 ng/ml. Finally, D2a10, with three sulfate groups, had 0.03 ± 0.05 ng/ml. D0a4/D2a0 was found in the largest concentration, with D0a0 and D0a6 having the next closest grouping. D0a10 also had a concentration greater than D2a4, D2a6, and D2a10. The latter three all had relatively low concentrations. In total, the mean concentration of the sulfated disaccharides was 190 ng/ml while the nonsulfated group was 28 ng/ml. Individual values can be found in Table 3.

**Table 3.** Chondroitin sulfate disaccharide concentrations (ng/ml) in CSF of 11 subjects

	01-01	01-02	01-03	01-04	01-05	01-06	01-07	01-08	02-01	02-02	02-03	Mean
D0a0	31.87	15.78	68.89	3.68	14.29	111.69	8.36	35.41	30.08	10.61	9.12	30.89
D0a4/D2a0	144.30	73.04	341.65	17.77	128.07	506.26	58.97	144.81	165.91	115.58	76.32	161.15
D0a6	22.74	7.50	51.83	1.05	21.39	34.13	16.59	53.31	54.48	8.59	8.64	25.48
D2a4	2.63	1.20	4.10	0.17	1.86	5.64	0.88	2.32	2.36	5.17	1.70	2.55
D2a6	1.82	0.61	2.70	0.02	1.97	5.95	0.94	2.32	2.20	2.61	7.28	2.58
D0a10	9.42	4.62	19.49	1.11	7.19	28.71	3.49	10.78	16.83	5.19	6.14	10.27
D2a10	0.03	0.00	0.00	0.00	0.00	0.14	0.00	0.04	0.07	0.04	0.01	0.03

Chondroitin sulfate disaccharide concentrations in CSF of 11 subjects [8 subjects from Site 1 (01-xx) and 3 subjects from Site 2 (02-xx)]. Three subject samples are not individually available for inclusion in this table but are incorporated into Figure 2.

### Heparan sulfate in ventricular CSF

**Figure 3.**
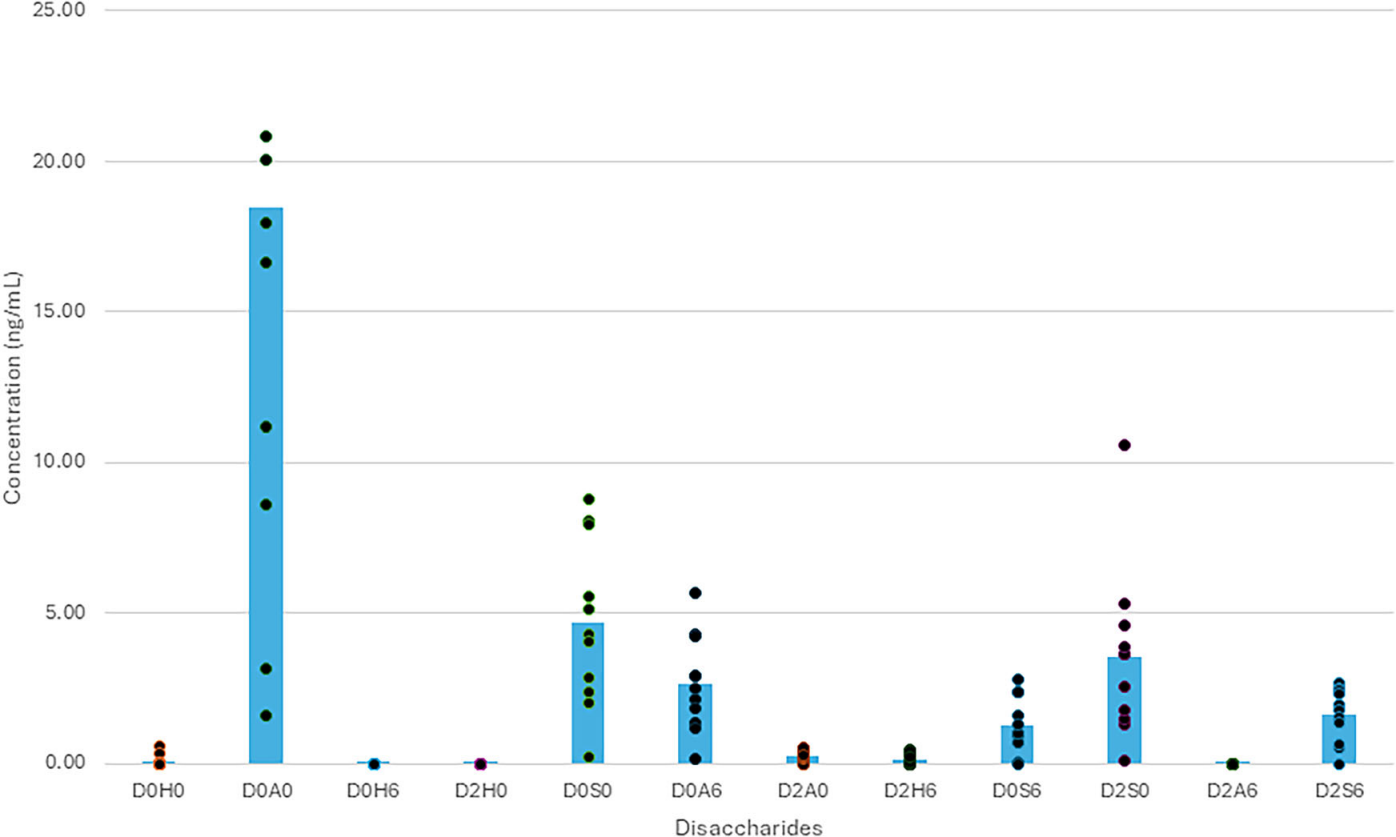
CSF heparan sulfate disaccharide concentration. Concentration of heparan sulfate disaccharides in CSF samples. Concentrations are measured in ng/ml with the D0A0 disaccharide having the highest mean concentration and statistically significant value compared with the rest of the disaccharides (*p* < 0.0005).

The CSF samples digested by heparinase I, II, and III (*n* = 21) showed a mean concentration of HS GAGs of 23 ± 17 ng/ml, which is a low value when compared with the normative results from lumbar punctures of 50 ± 16 ng/ml (Fig. 3; Hendriksz et al., 2015). Similar to the CS sGAGs, a single disaccharide, D0A0, which is not sulfated, showed the greatest concentration at 12 ± 10 ng/ml. Performing an ANOVA and post hoc test on the data set showed that the D0A0 disaccharide had statistically significant differences from the other disaccharides, with a *p*-value <0.0005 for all comparisons. For the additional disaccharides, the concentration of D0H0, which also is not sulfated, was 0.04 ± 0.09 ng/ml. For the disaccharides with one sulfate group, D0H6 had a concentration of 0.002 ± 0.004 ng/ml, D2H0 had 0.001 ± 0.003 ng/ml, D0S0 had 3.4 ± 2.9 ng/ml, D0A6 had 1.8 ± 1.3 ng/ml, and D2A0 had 0.18 ± 0.15 ng/ml. For the species with two sulfated groups, D2H6 had a concentration of 0.074 ± 0.13 ng/ml, D0S6 had 1.1 ± 4 ng/ml, D2S0 had 2.2 ± 1.6 ng/ml, and D2A6 had 0.002 ± 0.005. Finally, D2S3, which possesses three sulfated groups, had a concentration of 1.1 ± 0.7 ng/ml. In total, the average concentration of the sulfated disaccharides was 8.2 ng/ml, while the disaccharides without a sulfate group had a concentration of 11 ng/ml. This contrasts with the results in CSF digested by chondroitinase ABC, which had a higher concentration of sGAGs than non-sGAGs. A sample distribution of individual values can be found in Table 4.

**Table 4.** Heparan sulfate disaccharide concentrations (ng/ml) in CSF of 11 subjects

	01-01	01-02	01-03	01-04	01-05	01-06	01-07	01-08	02-01	02-02	02-03	Mean
D0H0	0.07	0.00	0.00	0.00	0.03	0.58	0.00	0.02	0.37	0.06	0.00	0.10
D0A0	20.05	3.16	36.08	1.60	20.82	33.99	8.62	17.98	33.36	16.62	11.20	18.50
D0H6	0.00	0.00	0.00	0.00	0.00	0.01	0.01	0.00	0.00	0.00	0.00	0.00
D2H0	0.00	0.00	0.00	0.00	0.00	0.00	0.00	0.01	0.00	0.00	0.00	0.00
D0S0	5.12	2.00	8.82	0.22	5.55	8.07	2.38	4.33	7.97	4.04	2.89	4.67
D0A6	4.28	1.40	5.71	0.17	2.91	4.26	1.19	2.16	2.90	2.48	1.83	2.66
D2A0	0.24	0.02	0.27	0.00	0.41	0.53	0.18	0.22	0.35	0.51	0.27	0.27
D2H6	0.00	0.00	0.00	0.00	0.33	0.48	0.06	0.04	0.31	0.40	0.23	0.17
D0S6	1.03	0.08	0.96	0.00	2.36	2.79	0.72	1.04	1.62	2.37	1.33	1.30
D2S0	3.62	1.32	4.58	0.10	10.61	5.33	1.50	1.80	3.62	3.86	2.58	3.54
D2A6	0.00	0.00	0.00	0.00	0.01	0.01	0.00	0.01	0.00	0.02	0.00	0.00
D2S6	1.99	0.54	2.66	0.00	2.50	2.45	0.66	1.78	1.57	2.34	1.36	1.62

Heparan sulfate disaccharide concentrations in CSF of 11 subjects [8 subjects from Site 1 (01-xx) and 3 subjects from Site 2 (02-xx)]. Three subject samples are not individually available for inclusion in this table but are incorporated into Figure 3.

### Plasma and urine sGAG levels

The levels of sGAGs found in both CS and HS plasma samples were greater on average than those found in the ventricular CSF. There were 18 unique plasma samples obtained, with some larger samples for a single time point being analyzed separately and then subsequently averaged. Plasma CS sGAGs were found to have a concentration of 7.4 µg/ml, which is an order of magnitude larger than the concentration found in the CSF. For HS, the concentration of sGAGs was found to be 0.23 µg/ml, which is also an order of magnitude larger than that in CSF samples.

For assessment of urine, there were a total of 12 unique samples, reflecting the Waiver of Consent constraints that did not allow for clinically unnecessary sample collection during every time epoch. The levels found in the urine samples were found to have a concentration of 3.1 µg/ml for CS and 1.7 µg/ml for HS, also greater than CSF levels.

### CSF and plasma sGAG concentration comparison

**Figure 4.**
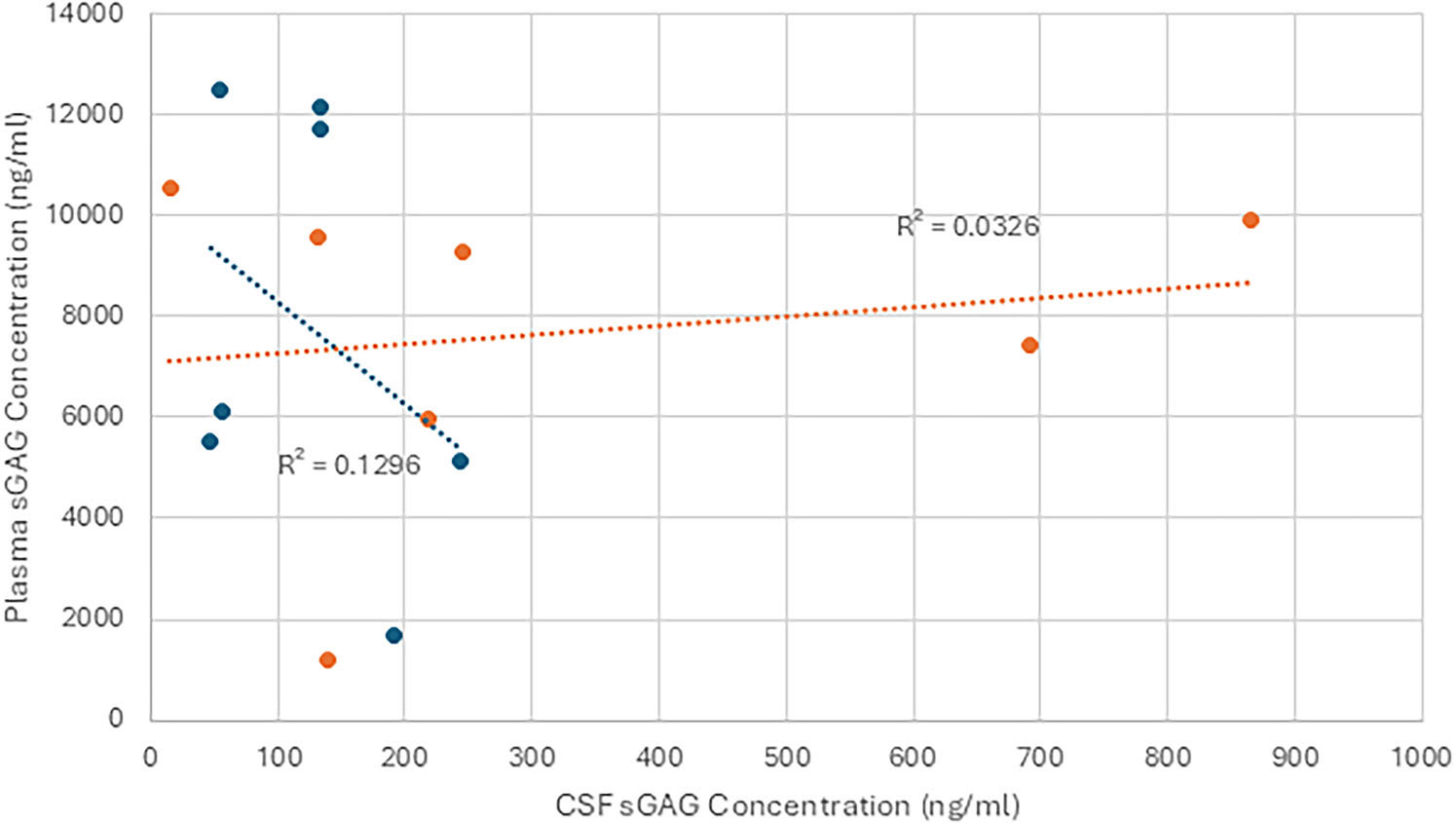
CSF versus plasma chondroitin sulfate concentration. Plasma and CSF sGAG concentrations (ng/ml) of chondroitin sulfate plotted against one another and separated into groups denoting isolated head trauma or multitrauma. No correlation was observed either within the groups or between the groups. The linear regression of each of the individual groups was also not found to be significant. Blue represents isolated head trauma, orange represents multitrauma.

**Figure 5.**
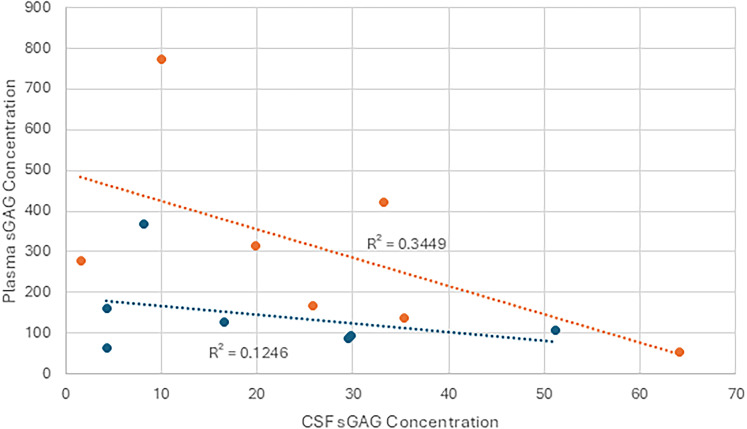
CSF versus plasma heparan sulfate concentration. Plasma and CSF sGAG concentrations (ng/ml) of heparan sulfate plotted against one another and separated into groups denoting isolated head trauma or multitrauma. No correlation was observed either within the groups or between the groups. The linear regression of each of the individual groups was also not found to be significant. While plasma levels on average were higher in multitrauma patients compared with those with isolated head injury, these differences were not significant. Blue represents isolated head trauma, orange represents multitrauma.

Comparing the levels of sGAGs in CSF and plasma highlights a difference in the concentrations (Figs. 4, 5). To further elucidate this difference and test whether a relationship is present, the concentrations were plotted against one another and separated into groups of samples from patients with isolated head trauma compared with those with multitrauma. With CS, Pearson’s correlation coefficient between isolated head trauma CSF and plasma sGAG values was found to be −0.36. This was not statistically significant as the *p*-value of the correlation coefficient was found to be 0.61. For multitrauma, Pearson’s correlation coefficient of 0.18 also was found to not be statistically significant with a *p*-value of 1.23. These data suggest no statistically significant correlation between CSF and plasma sGAG levels in either multitrauma or isolated head trauma in the CS samples.

For the HS samples, there also was no significant relationship between CSF concentrations and blood concentrations. Pearson’s correlation coefficient for isolated head trauma was −0.35 with a *p*-value of 0.63, and for multitrauma, the coefficient was −0.58, with a *p*-value of 0.15.

### Trauma-dependent effects

**Figure 6.**
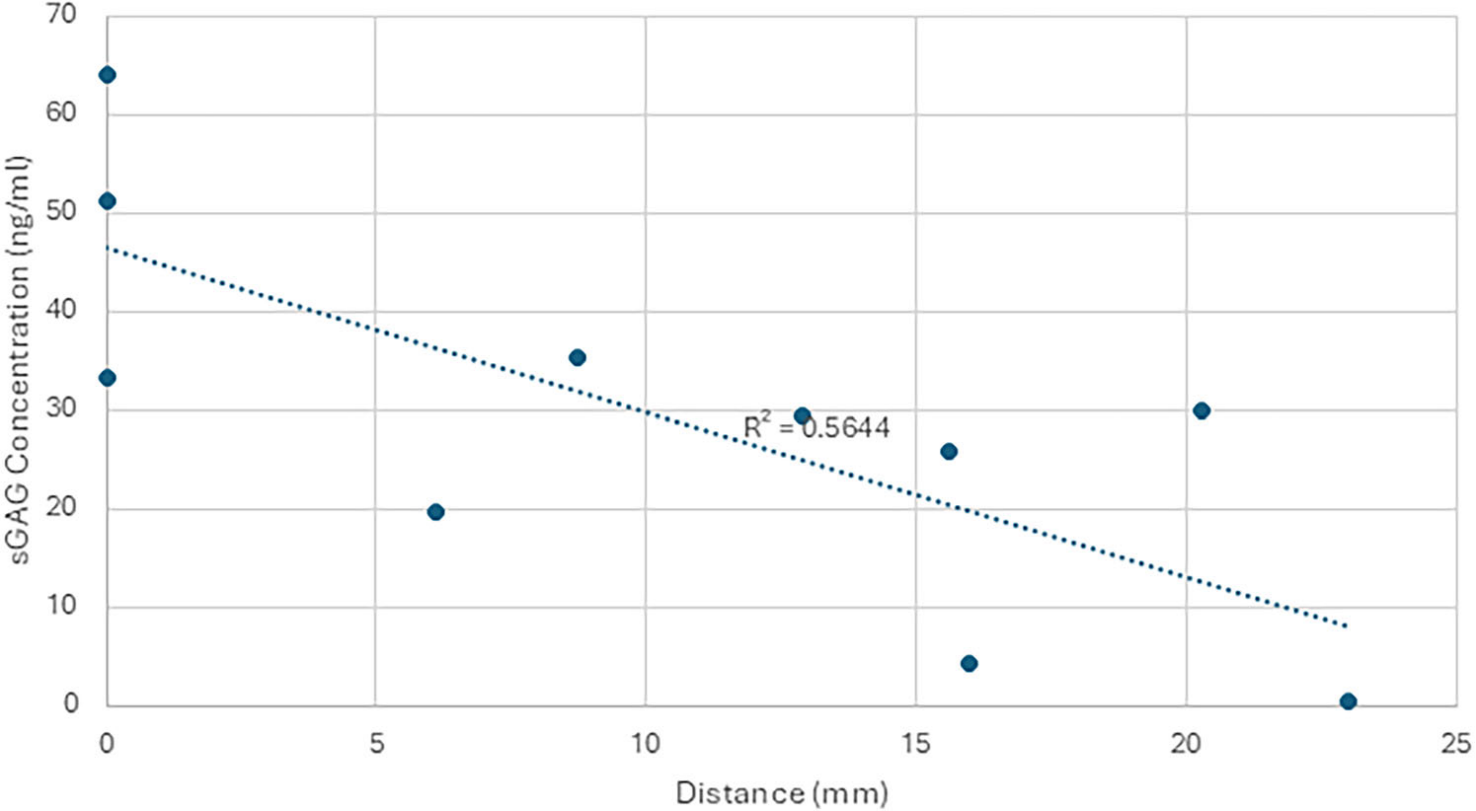
Distance from lateral ventricle compared with heparan sulfate sGAG concentration. Heparan sulfate concentration in CSF samples plotted against distance from the lateral ventricle where the external ventricular drain was placed. Pearson’s correlation was found to be −0.75 with a *p*-value of 0.048. This effect was not found in chondroitin sulfate CSF samples.

In both CS and HS sGAG concentrations, individual subjects displayed a wide range of values. In order to assess if there is a trauma-dependent effect the values were assessed against extracranial trauma, imaging load, ISS, distance from lateral ventricle, and age (Fig. 6).

There was no correlation between the concentrations CSF CS sGAGs to the degree of extracranial trauma, ISS, radiologic assessment of cerebral injury load, distance of injury to lateral ventricle, or patient age. Proximity of radiologically visible traumatic brain injury to the lateral ventricle had the strongest correlation, though still not significant with a *p*-value of 0.55. Similarly, no correlation was found between extracranial trauma, ISS, imaging load, or age and CSF HS sGAG concentrations. However, the concentration of HS sGAGs in the ventricular CSF was found to correlate with the distance of the visible injury from the lateral ventricle. Pearson’s correlation was found to be −0.75 with a *p*-value of 0.048.

## Discussion

This study describes sGAG levels in the ventricular CSF, blood, and urine of human patients with severe head trauma requiring ventricular CSF drainage for the management of intracranial pressure. Feasibility was demonstrated for the minimally invasive sampling of CSF (in the setting of an indwelling ventricular catheter for clinical care), blood, and urine; multicenter collaboration; and remote analysis of GAGs at a specialized center. The levels of CSF sGAGs were low relative to prior analyses of normal human lumbar CSF. There were borderline correlations between ventricular GAG levels and the proximity of traumatic lesions to the ventricles; however, large amounts of sGAGS were not found in the ventricular CSF or blood or urine after severe TBI, suggesting local rapid metabolism and clearance. The slight elevation seen when lesions are near the ventricle may suggest that some sGAGs may diffuse into ventricular fluid but that either sGAGs are not released or that they are rapidly metabolized. Since these were severe injuries with marked tissue disruption, it seems unlikely that sGAGs were not structurally disrupted by mechanical trauma, just as are other components of the cerebral parenchyma. However, the small sample size, the inherent variability of traumatic brain injury, and the natural variation in sGAG content and composition across individuals may have limited the power to detect local events as reflected in the current findings.

To the authors’ knowledge, this is the first report of GAG concentrations in human ventricular CSF. Ventricular CSF is not available from control patients due to the invasive nature of the sampling procedure. These samples were obtained from comatose trauma patients whose ventricular CSF was being drained to manage increased intracranial pressure. Despite the severity of the brain injury, the ventricular CSF GAG levels were lower than human control CSF obtained from lumbar puncture (Zhang et al., 2011; Hendriksz et al., 2015; Yu et al., 2019). Below, we consider two potential explanations for this finding.

Lumbar CSF has higher levels of protein and cells than ventricular CSF (Podkovik et al., 2020). This is thought to be a consequence of the proximity of ventricular CSF to the choroid plexus, where CSF is produced. In contrast, CSF collected from the lumbar space has diffused over the surface of the cortex and brain, providing a greater opportunity to pick up protein and cells extruded from these surfaces. The same may be true for GAGs. Experimental studies of cerebral edema have found that neutral and positively charged molecules can flow into ventricular CSF (Reulen et al., 1978) but molecules that are negatively charged at physiological pH, such as albumin and fluorescein isothiocyanate, flow to the subarachnoid CSF (Ohata and Marmarou, 1992). The negatively charged sGAGs therefore may not have flowed from the brain parenchyma to the ventricular CSF.

Another potential explanation for the low levels of ventricular CSF sGAGs in trauma patients is that sGAGs are not released into any CSF space under these conditions. In other words, after severe brain injury, dissolution of the extracellular matrix (Griffiths et al., 2020) may not result in significant spillage of matrix components into either the ventricular or subarachnoid CSF. Most of what is known regarding the catabolism of GAGs is based on studies of genetic defects in GAG catabolism. These defects form the bases for the human mucopolysaccharidoses such as Hunter's, Hurler's, and Sanfilippo diseases. In these disorders of GAG catabolism, neurons, glia, and vascular cells accumulate partially degraded GAGs that cannot be further broken down due to enzyme deficiency (Bigger et al., 2018). GAG components that are not catabolized are also stored in peripheral organs and excreted in the urine; however, there is no evidence that these GAGs arise from the brain rather than the peripheral organs. Lumbar CSF levels of GAGs are modestly elevated in these disorders (Zhang et al., 2011; Hendriksz et al., 2015). Therefore, there is not much evidence for substantial spillage of partially catabolized GAGs from the brain into CSF, blood, and urine in genetic disorders of GAG catabolism. It is not known at what rate brain GAGs are released by matrix metalloproteinases after human trauma (Abdul-Muneer et al., 2016; Glykys et al., 2017), nor the rate at which the released GAGs can be taken up by surviving neurons and glia. We speculate that after severe brain trauma, surviving neurons, glia, and perhaps vascular cells take up the majority of the GAGs released by matrix metalloproteinases, limiting GAG spillage into the CSF and bloodstream.

The modest correlations between HS and CS levels and the proximity of traumatic lesions to the ventricular surface suggest that spillage of sGAGs into ventricular CSF occurs in at least some circumstances. This relationship bears further investigation, because in our cohort, a certain quantity of blood was also present in the ventricular CSF of patients with lesions that were closest to the ventricular surface. The blood may have had higher sGAG levels (Figs. 3, 4), resulting in spurious CSF sGAG elevation. Alternatively, the presence of blood and sGAGs in the ventricular CSF in patients with lesions near the ventricular surface may arise independently from damage to the periventricular ECM and the ependymal lining of the ventricles. Normally, the fixed negative charges borne by intact sGAGs in the periventricular ECM should limit the diffusion of anionic molecules by Donnan exclusion (Helfferich, 1995; Epsztein et al., 2018), and the flux of sGAGs should be further reduced by the ependymal lining of the ventricles (Sarnat, 1995).

This study was limited by the number of subjects, especially in light of the variability of the age at the time of injury, traumatic injuries, and sGAG levels. In addition, full access to medical record data was only available for Site 1. Furthermore, enrollment was paused for 6 months during the COVID-19 pandemic and was limited thereafter by the reduction in motor vehicle usage and accident rates (Asaad et al., 2020). In the absence of COVID, we would expect an approximate 50% increase in the rate of enrollment over the course of this study. The study is also limited by a lack of controls; it is not ethical to obtain control human ventricular CSF. We focused on sGAGs, which are plentiful in the brain extracellular matrix, but there are many other matrix components that could also be studied (Karamanos et al., 2021). While this study eliminates one possibility for the fate of sGAGs released by matrix metalloproteinases after traumatic brain injury (transfer to CSF), it does not identify the path of further catabolism of the released sGAGs.

This pilot study will hopefully lay the groundwork for larger clinical studies by presenting the concentrations and distributions of the sGAGs and disaccharides present within the ventricular CSF and plasma in a two-center group of severely injured trauma patients. Addressing the mechanistic questions raised by this observational clinical study may best be accomplished using experimental preparations (Costine-Bartell et al., 2021) in which both intra- and extracellular sGAG levels can be analyzed after controlled subtypes of traumatic brain injury.

## Synthesis

Reviewing Editor: Jennifer Dulin, Texas A&M University Decisions are customarily a result of the Reviewing Editor and the peer reviewers coming together and discussing their recommendations until a consensus is reached. When revisions are invited, a fact-based synthesis statement explaining their decision and outlining what is needed to prepare a revision will be listed below. The following reviewer(s) agreed to reveal their identity: NONE.

The manuscript just needs one minor revision. Please see below.

Reviewer's comment:

The data has been extended as in they added tables with the individual values, which is not what I meant when I asked them to add individual points so we could see the spread of the data. I meant keep the bar graph, but add dots for each point on the bar. This should be a one-click change within your graphing software. Please make this change (with or without the table)
